# Context-dependent sublethal hazard of glyphosate to non-target predators revealed by functional response analysis

**DOI:** 10.1007/s10646-026-03179-5

**Published:** 2026-10-23

**Authors:** Jana Niedobová, Radek Michalko

**Affiliations:** 1 Department of Forest Ecology, Faculty of Forestry and Wood Technology, Mendel University in Brno, Zemědělská 3, Brno, 613 00 Czech Republic; 2 Research and Breeding Institute of Pomology Holovousy Ltd, Holovousy 129, Hořice, 508 01 Czech Republic

**Keywords:** Glyphosate, Ecotoxicology, Spiders, Predatory efficiency, Functional response, Ecotoxicological mechanisms

## Abstract

Glyphosate, one of the most widely used herbicides globally, is a significant environmental contaminant that may affect non-target organisms crucial to ecosystem services, including biological pest control. While glyphosate targets the shikimate pathway in plants, which is absent in animals, there is increasing evidence that glyphosate induces sublethal effects on arthropod predators. However, the factors modulating these effects remain unclear. Here, we investigated how physiological context, specifically pre-treatment with the broad-spectrum antibiotic tetracycline, commonly used to experimentally alter host associated microbial communities, modulates the sublethal hazard of glyphosate-based herbicide (GBH) on the predatory behavior of adult female *Pardosa* wolf spiders. Predation efficiency was measured using functional response assays across varying prey densities. Our results showed that GBH significantly reduced the spiders’ predation rates, confirming its sublethal toxicity. However, spiders pre-treated with antibiotics exhibited higher capture rates following GBH exposure compared to the GBH only group. These findings demonstrate that the sublethal hazard of GBH on predators is context-dependent and can be altered by antibiotic-sensitive processes (such as host-symbiont interactions). This study highlights the complexity of pesticide effects on ecosystem services and suggests that the functional response of non-target predators depends strongly on their physiological background.

## Introduction

Glyphosate is among the pesticides of greatest concern regarding its environmental impacts. It is the most widely used herbicide worldwide, formulated in more than 750 commercial products (Valavanidis 2018). Glyphosate-based herbicides (GBH) have been applied for over fifty years, and its use has steadily increased since the introduction of glyphosate-resistant crops in 1996 (Gill et al. 2017; Mayers et al. 2016), with approximately 700 thousand tonnes applied annually across 36 million km² of land (Maggi et al. 2019). The biochemical target of glyphosate is the shikimate acid pathway, present in plants and some microorganisms but absent in animals (Steinrücken and Amrhein 1980). Owing to this selectivity, glyphosate was long considered safe for non-target species, including vertebrates and invertebrates (Franz et al. 1997; Lee and Choi 2017). However, most environmental exposures involve commercial GBHs rather than glyphosate alone. These formulations contain surfactants and other co-formulants that may themselves exhibit biological activity or modify the toxicity of glyphosate through additive or synergistic interactions (Cedergreen 2014; Mesnage and Antoniou 2018). Consequently, it is often difficult to attribute observed biological effects solely to the active ingredient. Increasing evidence indicates that GBHs can induce sublethal effects in non-target arthropods, despite the primary biochemical target of glyphosate being absent in animals. Among these, spiders are increasingly recognised as a sensitive group, with GBH reported to alter locomotion, reproduction and predatory efficiency (Behrend and Rypstra 2018; Benamú et al. 2010; Evans et al. 2010; Griesinger et al. 2011; Lacava et al. 2021; Leccia et al. 2016; Niedobová et al. 2022; Wrinn et al. 2012). Spiders, as abundant generalist predators, play a key role in pest suppression and contribute to agricultural sustainability (Benamú et al. 2010; Keswani 2023; Michalko et al. 2019). Their abundance, ecological versatility, and sensitivity to agrochemicals make them ideal bioindicators and model organisms for studying sublethal pesticide effects (Pekár 2012). Among them, wolf spiders (Lycosidae) are particularly suitable models for studying GBH effects, as their ground-dwelling behavior increases exposure to herbicide residues in agroecosystems (Niedobová et al. 2022). Although several studies report reductions in predatory performance under GBH exposure (Behrend and Rypstra 2018; Lacava et al. 2021; Niedobová et al. 2022; Rittman et al. 2013), the mechanisms underlying these behavioral changes remain unclear, especially given that the primary biochemical target of glyphosate does not occur in animals.

In recent years, accumulating evidence has indicated that glyphosate can influence ecosystem functions by altering microbial communities (Ruuskanen et al. 2023). Although glyphosate inhibits plant 5-enolpyruvylshikimate-3-phosphate synthase, it also disrupts microbial communities in soil, host-associated microbial communities, and other habitats of both invertebrates and vertebrates (Barnett et al. 2022; Blot et al. 2019; Del Castilo et al. 2022; Evalen et al. 2024; Gao et al. 2023; Mesnage and Antoniou 2020; Mesnage et al. 2021; Muñoz et al. 2025; Tang et al. 2020). Such findings have led to the hypothesis that some of glyphosate’s sublethal effects on animals could be mediated indirectly, at least in part, through changes in host-associated microbial communities. In insects, microbial associates are increasingly recognised as determinants of fitness-related processes (Engel and Moran 2013; Heider et al. 2025, Shamjana et al. 2024). They can also degrade or transform toxic compounds, alter bioavailability, and even contribute to pesticide resistance (Itoh et al. 2018; Peterson 2024). Glyphosate-induced disruption of gut microbiota has been documented in pollinators, with consequences for resilience, immunity and behaviour (Motta et al. 2018). Although comparable mechanisms have not yet been established in spiders, these studies collectively suggest that microbial associates may influence how arthropods respond to pesticide exposure. Spiders, like other arthropods, harbour diverse host-associated microbial communities comprising both gut-associated bacteria involved in nutrient metabolism and tissue-resident microbial symbionts (Wu et al. 2021; Tyagi et al. 2021; Zhang et al. 2018). Both microbial compartments have been implicated in host physiology and fitness, although their respective roles in mediating responses to pesticides remain poorly understood.

Among these tissue-resident microbes, intracellular bacterial endosymbionts such as *Wolbachia*, *Cardinium*, *Rickettsia*, *Spiroplasma*, and *Rickettsiella* (Zhang et al. 2018) are increasingly recognised as modulators of host physiology and behavior (Goodacre and Martin 2012; Beydizada et al. 2023). These symbionts can influence traits, such as nutrition, immunity, stress tolerance, and pesticide resistance (Řezáč et al. 2025a; 2025b).

In laboratory research, tetracycline is widely used to experimentally perturb host-associated microbial communities in order to investigate microbial functions. In spiders, its application has been primarily focused on eliminating tissue-resident bacterial endosymbionts such as *Wolbachia*, *Cardinium*, *Spiroplasma* and *Rickettsia* (Goodacre 2011; Gotoh et al. 2007; Mazzetto et al. 2015; Li et al. 2018), whereas in other arthropods tetracycline has become an established experimental tool for inducing gut microbiota dysbiosis, enabling investigations of microbial contributions to host physiology, metabolism, immunity, and fitness (Raymann et al. 2018; Li et al.^ 2026^).

Given that specific microbial changes cannot be definitively inferred without direct molecular data, the use of tetracycline provides a well-established means of experimentally perturbing the internal physiological background to assess the context-dependency of pesticide toxicity.

Despite this growing body of evidence, the mechanisms underlying sublethal hazard of glyphosate and GBH on predators remain unresolved. Here, we combined a behavioral ecotoxicological approach with a well-established broad-spectrum antibiotic treatment to test whether perturbation of antibiotic-sensitive host-associated microbial communities and associated physiological processes modifies the sublethal effects of a GBH on a non-target predator. Adult *Pardosa* spiders were pre-exposed to tetracycline and subsequently treated with the GBH Roundup^®^ Klasik Pro. Rather than aiming to identify the underlaying mechamism or characterize microbial communities directly, our goal was to test whether an antibiotic-induced alternation of the physiological state modifies the sublethal effects of GBH on spider predation. Predatory performance was assessed using functional response analysis, which measures the per capita number of prey killed at different prey densities over time (Benhadi-Marín et al. 2019) and provides a mechanistic framework linking predator behaviour to fluctuating prey populations in the field (Benhadi-Marín et al. 2018). Because both GBH and antibiotics can independently influence host physiology, their combined effects cannot be assumed to be simply additive. In ecotoxicology, simultaneous exposure to multiple stressors may generate additive, synergistic or antagonistic responses depending on the underlying physiological mechanisms. If GBH-induced effects are mediated through antibiotic-sensitive microbial processes, pre-treatment with tetracycline could either exacerbate or attenuate the behavioural consequences of glyphosate exposure.

In this study, we hypothesized that: (i) GBH exposure reduces the predatory efficiency of wolf spiders, reflected in lower capture rates across prey densities; (ii) Antibiotic pre-treatment may independently affect predatory behaviour by altering host-associated microbial communities and associated physiological processes. Consequently, the combined exposure to tetracycline and the GBH was expected to produce non-additive effects (synergistic or antagonistic) on predatory efficiency; and (iii) Functional response analysis provides a robust framework to detect such context-dependent hazards, with both GBH and antibiotic treatments expected to influence short- and medium-term prey capture dynamics.

## Materials and methods

### Collection of studied species and maintenance

Adult female spiders of comparable prosoma size (width 1.8–1.9 mm, length 2.1–2.2 mm) belonging to the *Pardosa lugubris* group (*N* = 240) were hand-collected on 9 May 2024 at a chemically untreated forest near Drnovice, Czech Republic (49.2782311 N, 16.9368753E). Each specimen was placed individually into 1.5 ml microcentrifuge tubes with a pierced lid to ensure ventilation. The *Pardosa lugubris* group in Central Europe comprises two morphologically similar and co-occuring species, *Pardosa lugubris* (Walckenaer 1802) and *Pardosa alacris* (Koch 1833). For practical reasons, we refer to them collectively as the *P. lugubris* group, since females of these two species cannot be distinguished morphologically (Nentwig et al. 2024). The sampling locality is managed without herbicide or insecticide applications, providing a population with minimal prior agrochemical exposure and thus a realistic baseline for assessing sublethal glyphosate effects. The sampling date (early May) coincides with the main period of glyphosate applications in Central European orchards, when epigeic wolf spiders are active on the soil surface and are likely to encounter both fresh spray and surface residues in the field.

In the laboratory, spiders were transferred into larger plastic tubes (15 mL) and randomly assigned to one of four experimental groups: Control, GBH, ATB (tetracycline), and ATB + GBH. Each treatment included 60 individuals. Spiders allocated to the ATB treatments were provided ad libitum to a tetracycline solution on a moistened cotton swab, whereas the remaining spiders received only water. All individuals were maintained at 19 °C under a natural photoperiod (15 L:9D), corresponding to the natural day length in the Czech Republic during the experimental period (mid-May).

To minimize the reduction of tetracycline efficacy through photodegradation, a freshly moistened cotton swab with tetracycline solution (or water for glyphosate and control) was supplied each evening. All spiders were fed ad libitum for five days with fruit flies *Drosophila melanogaster* (Meigen, 1830). Thereafter, they were starved for an additional five days to ensure a comparable hunger level across individuals. During the starvation period, tetracycline solution or water was provided daily in the same manner.

### Preparation of tetracycline and glyphosate product

The rationale for using tetracycline to experimentally alter host-associated microbial communities is described in the Introduction. Here, we provide only the methodological information relevant to the experimental treatment and dosage. Although we did not directly validate bacterial community changes in *Pardosa*, tetracycline has previously been shown in Arachnida to alter bacterial symbiont infection patterns or community composition, including in spiders (Goodacre et al. 2009) and ticks (Zhang et al. 2024; Guizzo et al. 2017). The tetracycline mode of action is based on inhibition of protein synthesis (Li et al. 2014). For the experiment, tetracycline (Sigma-Aldrich) was diluted in distilled water to a final concentration of 0.3 mg/mL following Mazzetto et al. (2015). Similar tetracycline concentrations have been widely employed in studies of arthropod endosymbionts to reduce or eliminate bacterial infections while maintaining host viability, thereby allowing the assessment of endosymbiont-associated phenotypic effects (Mazzetto et al. 2015; Gotoh et al. 2007; Li et al. 2018).

The GBH Roundup^®^ Klasik Pro, containing 360 g glyphosate/L, was diluted at 3 mL product per 100 mL water. This dilution corresponds to 30 mL product per litre of water and reproduces the manufacturer’s recommended field application rate of 6 L product applied with 200 L water per hectare. Based on the declared active ingredient content, this dilution corresponds to 10.8 g glyphosate a.i./L when expressed relative to the water volume used for dilution. This value refers to the concentration of glyphosate in the spray mixture before application and should not be interpreted as an environmental concentration in runoff, soil pore water, or surface water. The corresponding nominal field application rate was 2.16 kg glyphosate a.i./ha, representing a label-rate direct spray exposure scenario relevant to orchard and vineyard applications.

### Laboratory testing

Spiders were individually transferred into chambers and treated with a Potter spray tower (Burkard Manufacturing, Rickmansworth, Hertfordshire, England) at 50 kPa, delivering a field-equivalent deposit of 2 mg/cm² ± 10%. This spray volume was calibrated to reproduce the surface loading expected when 200 L/ha of spray solution are applied uniformly over the treated area, thereby approximating field-equivalent coverage of soil and low vegetation. Based on the glyphosate concentration in the working solution, this deposition corresponds to 0.0216 mg a.i./cm². After application, each spider was placed in a clean Petri dish (5 cm diameter) with a moistened cotton swab to provide water. Spiders were allowed to settle for 30 min before testing.

To evaluate treatment effects on the functional response, each of the four treatment groups (Control, GBH, ATB, and GBH + ATB) was further divided into subgroups according to prey density. Spiders were offered one of five densities of *Drosophila* flies (1, 3, 6, 9, or 12 individuals). Twelve spiders were tested at each density. Each Petri dish was labeled with the treatment code, spider identity, and assigned prey density.

Killed prey was monitored at two temporal scales: short term (1st – 5th hour) and medium term (2nd – 5th day). Each spider was exposed to the same prey density throughout the entire observation period. To maintain constant prey availability, killed flies were immediately replaced with live ones after each count. In the short-term assay, the number of prey killed was recorded hourly for five consecutive hours (five records per spider). In the medium-term assay, prey consumption was recorded daily over four consecutive days using the same spiders (four records per spider). These two temporal windows were selected to capture both acute behavioral changes following exposure and persistent sublethal effects that may be relevant for daily predation in the field.

Spiders that had molted within three days prior to Potter tower spraying, during the experiment, or within one day after the experiment ended were excluded from the analysis, because spiders generally do not feed before molting (Korenko et al. 2016; Niedobová et al. 2014). In total, 25 spiders were excluded. The final sample sizes used in the analyses were: GBH (*N* = 48), ATB (*N* = 58), GBH + ATB (*N* = 58), and Control (*N* = 51).

### Statistical analysis

All analyses were performed within the R environment (R Core Team 2023). Mortality among treatments was compared by the Cox Proportional Hazard test (CPH).

We modeled the capture rate along prey density as a mean capture rate across the short term (1st – 5th hour) and medium term (2nd – 5th day). We used the generalized linear models (GLM) with Gamma error structure and inverse link function (GLM-g). We further inverse transformed the explanatory variable prey density as the mean capture rate seemed to increase asymptotically with prey density in all treatments (Řezáč et al. 2010; Michalko and Košulič 2016). To investigate the effect of treatment on the capture rate, we added the effect of treatment and the interaction between treatment and prey density in the linear predictor so the initial model was in R syntax as follows: treatment*I(1/prey density). “I()” is a specific command that tells R to treat everything inside the parentheses as an ordinary mathematical expression. The non-significant terms were excluded according to their significance and the rule of marginality. The pairwise comparisons among factor levels were performed using the method called “treatment contrasts”, as implemented in the summary() function, by changing the reference level. No adjustment of P-values was applied because these contrasts represented planned comparisons.

## Results

### Mortality

The mortality of *Pardosa* spiders did not differ significantly among the treatments (CPH, χ^2^_3_ = 5.0, *P* = 0.172).

### Effects of treatments on capture rate

**Fig. 1:**
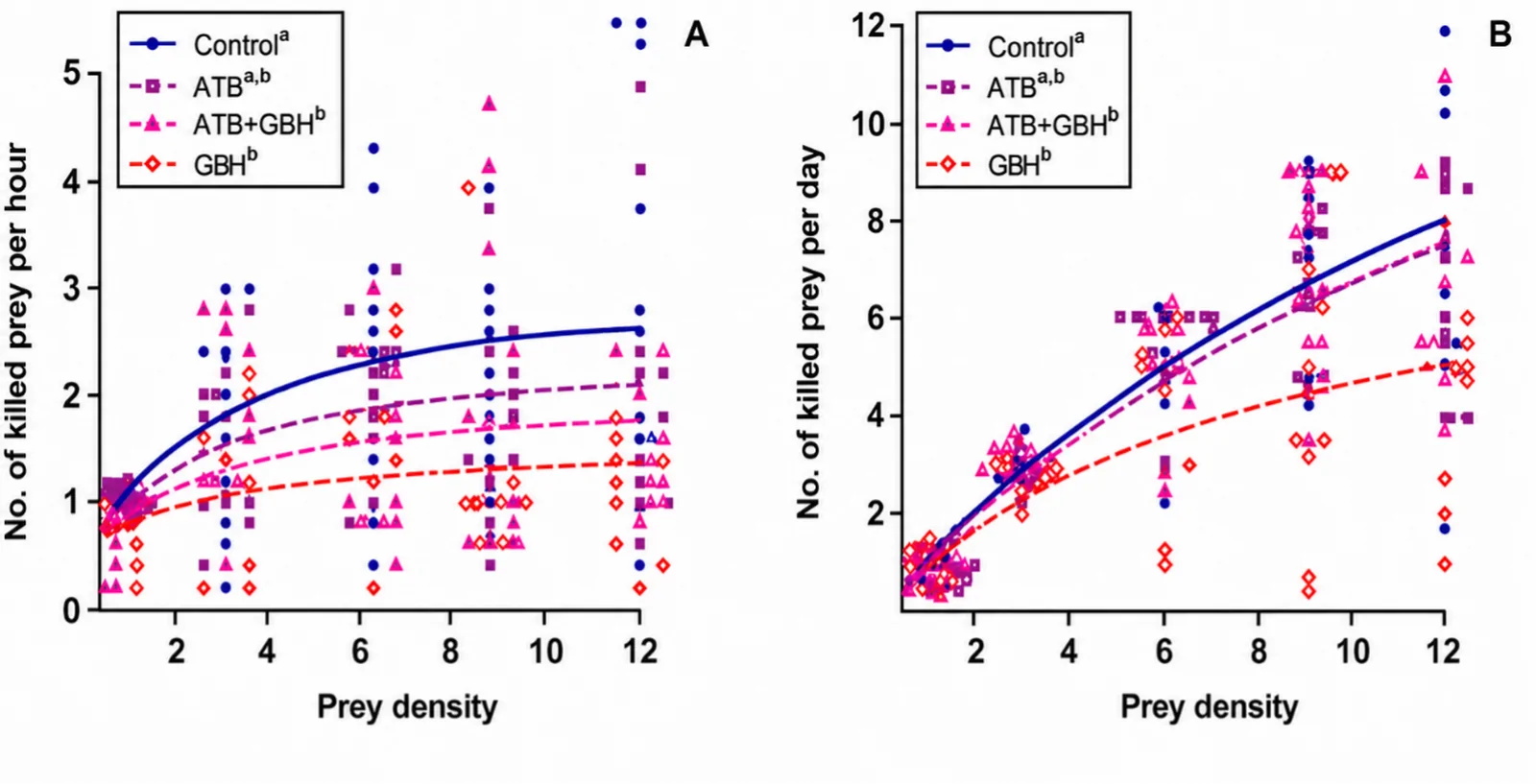
Short term (**A**; prey capture recorded hourly during the first 5 h after exposure) and medium term (**B**; prey capture recorded daily from the 2nd to the 5th day after exposure) functional response of *Pardosa* spiders to the densities of *Drosophila* flies after exposure to one of four treatments, namely control, antibiotics (ATB), glyphosate-based herbicide (GBH), and antibiotics and glyphosate-based herbicide (ATB + GBH). The points are individual measurements, and the lines are the estimated models. Small noise was added to the points to show the data structure. Different superscripts in the legend show statistical difference

In a short term, the number of killed prey significantly increased with increasing prey density (GLM-g, F_1,199_ = 49.0, *P* < 0.001; Fig. 1A). The treatment additively influenced the capture rate (GLM-g, F_3,200_ = 8.8, *P* < 0.001; Fig. 1A). Spiders in the control treatment had the highest capture rate (contrasts, *P* < 0.002; Table 1) which was not significantly different from the spiders in ATB treatment (contrasts, *P* = 0.055; Table 1). The spiders from the ATB + GBH treatment had lower capture rate than then the control treatment (contrasts, *P* = 0.002) but not different than the ATB treatment (contrasts, *P* = 0.167; Table 1). The lowest capture rate had the spiders from the GBH treatment (contrasts, *P* < 0.046; Table 1).

**Table 1:** Pair-wise comparisons of short term (prey capture recorded hourly during the first 5 h after exposure) functional response of *Pardosa* spiders to the densities of *Drosophila* flies after exposure to one of four treatments, namely control, antibiotics (ATB), glyphosate-based herbicide (GBH), and antibiotics and glyphosate-based herbicide (ATB + GBH)

Compared treatments		t-value	*P*-value
Control vs. ATB	1.933		0.055
Control vs. GBH	4.661		< 0.001
Control vs. GBH + ATB	3.195		0.002
ATB vs. GBH	3.215		0.002
ATB vs. GBH + ATB	1.386		0.167
GBH vs. GBH + ATB	-2.011		0.046

In a medium term, the capture rate increased with increasing fly densities (GLM-g, F_1,190_ = 916.8, *P* < 0.001; Fig. 1B) and the capture rate was additively affected by a treatment (GLM-g, F_3,191_ = 12.2, *P* < 0.001; Fig. 1B). The lowest capture rate had spiders from the GBH treatment (contrasts, *P* < 0.001; Table 2) while other treatments did not differ significantly (contrasts, *P* > 0.423; Table 2).

**Table 2:** Pair-wise comparisons of long term (prey capture recorded daily from the 2nd to the 5th day after exposure) functional response of *Pardosa* spiders to the densities of *Drosophila* flies after exposure to one of four treatments, namely control, antibiotics (ATB), glyphosate-based herbicide (GBH), and antibiotics and glyphosate-based herbicide (ATB + GBH)

Compared treatments		t-value	*P*-value
Control vs. ATB	0.801		0.424
Control vs. GBH	5.329		< 0.001
Control vs. GBH + ATB	0.461		0.645
ATB vs. GBH	4.668		< 0.001
ATB vs. GBH + ATB	-0.327		0.774
GBH vs. GBH + ATB	-4.890		< 0.001

## Discussion

### Mortality

This result is consistent with most previous studies reporting predominantly sublethal effects of GBH in spiders (Behrend and Rypstra 2018; Benamú et al. 2010; Lacava et al. 2021; Niedobová et al. 2019, 2022). However, Evans et al. (2010) reported reduced long-term survival of *Pardosa milvina* following residual exposure, indicating that lethal effects may occur under specific exposure conditions.

### Effects of treatments on capture rate

We confirmed the first hypothesis that Roundup^®^ Klasik Pro exposure reduces the predatory efficiency of *Pardosa* spiders, reflected in lower capture rates across prey densities. Two previous studies focused on the effect of Roundup^®^ Klasik Pro on the predatory behavior of *Pardosa* spiders, though the results were inconsistent (Niedobová et al. 2022, 2019). Only the second study confirmed a reduction in predatory activity, both in the short term, (during the first four hours) and in the long term (over the subsequent four days). However, both studies used a constant prey density, which limits their ability to capture the complexity of predator-prey dynamics. In contrast, our experiment, which involved varying prey density and a large number of similarly sized female spiders, allowed for a more precise examination of Roundup^®^’s Klasik Pro effect on functional response.

The reduction in capture rate observed here is consistent with several studies reporting sublethal effects of GBH on wolf-spider foraging, although the direction and magnitude of the response vary considerably among species, prey types, exposure scenarios, and commercial formulations. In *Pardosa agricola*, contact with fresh or one-day-old residues of Roundup Biaktiv did not significantly affect prey consumption or other measured behaviours (Michalková and Pekár 2009). By contrast, exposure to a GBH altered prey capture differently in two co-occurring wolf spiders: *Tigrosa helluo* oriented towards and subdued prey more rapidly, whereas *Pardosa milvina* required more lunges to capture prey without a corresponding change in capture timing (Rittman et al. 2013). Similarly, GBH exposure reduced the functional response of *Hogna cf. bivittata* towards caterpillars and ants but not crickets, indicating that effects may also depend on prey identity and the demands associated with prey subjugation (Lacava et al. 2021). Longer-term responses may even differ in direction, as exposure of *P. milvina* during earlier life stages increased subsequent activity and prey capture success (Behrend and Rypstra 2018). Together, these contrasting findings indicate that the effects of GBH on wolf-spider predation are not uniform but depend on the spider species, prey characteristics, life stage, exposure route and duration, and the composition of the tested formulation. Our results therefore provide evidence specifically for a direct field-rate spray exposure to Roundup^®^ Klasik Pro and should not be interpreted as demonstrating an equivalent effect of glyphosate alone or of all glyphosate-based formulations.

Beyond wolf spiders, recent reviews and meta-analyses indicate that herbicides can reduce predation and other performance traits of arthropod natural enemies, even when acute mortality is relatively low (Pinheiro et al. 2023; Zilnik et al. 2023). Spiders were among the less sensitive groups in terms of herbicide-induced mortality, but predatory efficacy was nevertheless significantly reduced across natural enemies, emphasizing that survival alone may underestimate ecologically relevant impacts on biological control. These broader syntheses therefore support the ecological relevance of the behavioural changes observed in the present study.

Similar alterations in feeding-related behaviour have also been documented in a wide range of non-predatory organisms exposed to glyphosate or GBH. In non-predatory organisms, glyphosate exposure has been associated with reduced food consumption, impaired food preference and altered foraging decisions, often accompanied by changes in energy metabolism. For example, glyphosate altered feeding preferences and reduced food intake in zebrafish larvae, leading to impaired energy allocation and decreased survival during the critical first-feeding stage. Likewise, sublethal exposure in honey bees reduced sucrose responsiveness and impaired reward-associated learning, potentially affecting foraging efficiency despite the absence of obvious changes in foraging activity (Herbert et al. 2014). In *Drosophila*, Roundup exposure has been linked to reduced glucose levels and was suggested to decrease overall food consumption through disruption of metabolic homeostasis (Strilbytska et al. 2022). Together, these studies indicate that glyphosate and GBH can influence feeding behaviour through multiple physiological pathways, including changes in sensory perception, cognition and energy metabolism. Our results extend this emerging pattern to active arthropod predators by demonstrating that GBH also impairs prey capture efficiency, suggesting that sublethal effects of GBH may propagate across trophic levels not only by affecting herbivores and detritivores, but also by disrupting predator–prey interactions.

We also confirmed the second hypothesis that antibiotic treatment alters the behavioral response of spiders to GBH. Specifically, we found that ATB tetracycline pre-treatment attenuated the negative effect of Roundup^®^ Klasik Pro on predatory performance. Although tetracycline treatment alone showed only a non-significant trend towards reduced prey capture efficiency, this observation may indicate that antibiotic-sensitive host-associated microbial communities, or other physiological processes affected by tetracycline, contribute to predatory performance independently of herbicide exposure. Because we did not directly characterize the microbiota, this interpretation should be regarded as a hypothesis requiring future experimental validation. This interpretation is consistent with growing evidence that spiders harbour diverse host-associated microbial communities (Zhang et al. 2018; Řezáč et al. 2025a; 2025b, Goodacre 2011; Hu et al. 2019). Most studies have focused on the impact of specific intracellular bacterial groups on spider physiology and behavior. Symbionts like *Wolbachia*, *Rickettsia*, *Spiroplasma* and *Cardinium* have been shown to influence aspects such as fecundity and sex ratio (Goodacre 2011; Goodacre et al. 2009; Gunnarsson et al. 2009). For instance, tetracycline treatments, which were focused on *Wolbachia* infections, resulted in a more balanced sex ratio in *Erigone atra*, and similarly, it has been observed that endosymbiotic bacterial infections modulate dispersal behavior in spiders (Goodacre et al. 2009). *Erigone atra* females infected with *Rickettsia* exhibited a lower frequency of the “tiptoeing” posture essential for ballooning, whereas individuals cured of the infection by tetracycline treatment resumed normal dispersal behaviour (Goodacre et al. 2009).

However, little is known about how microbiome alterations affect spiders exposed to agrochemicals. Evidence from insects and other arthropods indicates that host-associated microbial communities can substantially modify pesticide susceptibility. Gut-associated microbes may directly degrade or transform toxic compounds or stimulate host detoxification pathways, thereby modulating pesticide toxicity and contributing to resistance (Jaffar et al. 2022; Peterson 2024). Although comparable mechanisms remain largely unexplored in spiders, these findings suggest that microbiome-mediated modulation of pesticide toxicity may represent a widespread phenomenon among arthropods. We can find only a scarce evidence, that endosymbiontsincluding *Mycoplasmata*, *Wolbachia*, and *Rickettsia* has been shown to drive insecticide resistance in *Philodromus cespitum* (Řezáč et al. 2025a), and similarly, studies in insects have demonstrated that microbial symbionts can mediate pesticide susceptibility through effects on detoxification and host physiology (Peterson 2024). Recent studies have shown that *Wolbachia* can modulate pesticide resistance in other members of Arachnida, such as the two-spotted spider mite (*Tetranychus urticae*). Ye et al. (2024) demonstrated that *Wolbachia* alleviates pesticide susceptibility in these mites through enhanced host detoxification pathways, which suggests a similar mechanism may be present in spiders. In contrast, our study revealed that spiders pre-treated with tetracycline exhibited higher capture rates under GBH exposure compared to GBH-only group, suggesting that antibiotic-sensitive processes can attenuate sublethal herbicide effect. One plausible explanation for this result is that glyphosate exerts part of its toxicity indirectly through microbiome disruption (Motta et al. 2018). Therefore, spiders with altered or reduced microbiomes following antibiotic treatment may have been less affected because this indirect pathway was perturbed.

Another possible explanation is that the “microbiota-gut–brain axis”, as proposed by Matsuzaki et al. (2023), could also play a role in modulating behavior. If GBH interferes with this signalling, spiders with an antibiotic-altered internal state may be less susceptible to behavioral impairments typically induced by the herbicide. While the primary mechanism of action explored in our study relates to the inhibition of bacterial symbionts, previous research has shown that tetracycline may also have direct antioxidant effects under low concentrations, potentially mitigating oxidative stress (Kładna et al. 2012; Zhou 2020). Although these effects have not been directly measured in our spider model, such properties suggest that direct physiological interactions between tetracyclines and oxidative stress pathways could also contribute to the observed behavioural responses following GBH exposure.

Finally, we confirmed the third hypothesis that functional response analysis provides a robust mechanistic framework to detect how GBH and antibiotic treatments alter predatory efficiency in spiders, and to capture potential microbiome-related modulation of these sublethal effects by internal physiological factors. While previous studies have primarily used sequencing-based approaches to characterize microbiome alterations following pesticide exposure (Řezáč et al. 2025a; 2025b), Matsuzaki et al. (2023) highlight that these methods alone cannot determine whether the observed microbial shifts have functional consequences for the host. As Matsuzaki et al. (2023) emphasize, functional analyses are crucial to clarify how microbiome disruption translates into physiological or behavioral changes. Our results confirm that *Pardosa* spiders exhibited a type II functional response in both short- and medium-term assays, with significant reductions in capture rates under GBH and antibiotic in combination with GBH treatments. Functional response of type II is typical for spiders, characterized by a decelerating asymptotic curve and a declining proportion of prey killed at higher prey densities, being typical for spiders (Riechert and Lockley 1984).

These findings highlight that antibiotic-sensitive processes can significantly influence trophic interactions and ecosystem services, particularly the natural pest control provided by beneficial predators. Our results suggest that internal physiological background, probably the microbiome status of non-target organisms should be explicitly considered in pesticide risk assessments. Alternation to these systems may undermine ecological functions that are essential for maintaining sustainable agricultural practices and enhancing biodiversity. Consequently, future pesticide regulation and management strategies should account for the indirect, microbiome-mediated effects on ecosystem health to safeguard critical ecological services.

## Conclusions

This study demonstrates that GBH exposure significantly reduces the predatory efficiency of *Pardosa* spiders, with sublethal effects observed across varying prey densities. Crucially, pre-exposure to tetracycline, an antibiotic known to affect microbial symbionts, mitigated these negative effects, suggesting that antibiotic sensitive processes (such as host-microbe interactions) play a role in modulating pesticide toxicity. Our findings highlight the potential of these internal physiological factors in influencing predator–prey interactions and the effectiveness of biological control in agroecosystems. This research underscores the importance of considering context-dependent hazards in future pesticide risk assessments, as changes in the internal state of organisms could compromise the ecosystem services provided by beneficial invertebrates. Further investigation into the specific role of host-associated microbes in pesticide responses will improve our understanding of their impact on natural pest control and guide more sustainable pest management practices. 

## Data Availability

The datasets generated during the current study are available in the figshare repository at: (https://figshare.com/articles/dataset/Dataset_Pardosa_predatory_activity_affected_by_glyphosate_commercial_products/14958042).
